# Economic evaluations of maternal health interventions: a scoping review

**DOI:** 10.12688/f1000research.76833.1

**Published:** 2022-02-24

**Authors:** Katherine E. Eddy, Alexander Eggleston, Sher Ting Chim, Rana Islamiah Zahroh, Elizabeth Sebastian, Chloe Bykersma, Steve McDonald, Caroline S. E. Homer, Nick Scott, Doris Chou, Olufemi T. Oladapo, Joshua P. Vogel

**Affiliations:** 1 Maternal, Child and Adolescent Health Program, Burnet Institute, Melbourne, Australia; 2 Faculty of Medicine, Nursing and Health Sciences, Monash University, Melbourne, Australia; 3 Gender and Women's Health Unit, Centre for Health Equity, Melbourne School of Population and Global Health, University of Melbourne, Melbourne, Australia; 4 School of Public Health and Preventive Medicine, Monash University, Melbourne, Australia; 5 UNDP/UNFPA/UNICEF/WHO/World Bank Special Programme of Research, Development and Research Training in Human Reproduction (HRP), Department of Sexual and Reproductive Health and Research, World Health Organization, Geneva, Switzerland

**Keywords:** Economic evaluation, cost-effectiveness, cost-utility, cost-benefit, health economics, maternal health, maternal interventions

## Abstract

***Background***

Evidence on the affordability and cost-effectiveness of interventions is critical to decision-making for clinical practice guidelines and development of national health policies. This study aimed to develop a repository of primary economic evaluations to support global maternal health guideline development and provide insights into the body of research conducted in this field.

***Methods***

A scoping review was conducted to identify and map available economic evaluations of maternal health interventions. We searched six databases (NHS Economic Evaluation Database, EconLit, PubMed, Embase, CINAHL and PsycInfo) on 20 November 2020 with no date, setting or language restrictions. Two authors assessed eligibility and extracted data independently. Included studies were categorised by subpopulation of women, level of care, intervention type, mechanism, and period, economic evaluation type and perspective, and whether the intervention is currently recommended by the World Health Organization. Frequency analysis was used to determine prevalence of parameters.

***Results***

In total 923 studies conducted in 72 countries were included. Most studies were conducted in high-income country settings (71.8%). Over half pertained to a general population of pregnant women, with the remainder focused on specific subgroups, such as women with preterm birth (6.2%) or those undergoing caesarean section (5.5%). The most common interventions of interest related to non-obstetric infections (23.9%), labour and childbirth care (17.0%), and obstetric complications (15.7%). Few studies addressed the major causes of maternal deaths globally. Over a third (36.5%) of studies were cost-utility analyses, 1.4% were cost-benefit analyses and the remainder were cost-effectiveness analyses.

***Conclusions***

This review provides a navigable, consolidated resource of economic evaluations in maternal health. We identified a clear evidence gap regarding economic evaluations of maternal health interventions in low- and middle-income countries. Future economic research should focus on interventions to address major drivers of maternal morbidity and mortality in these settings.

## Introduction

An estimated 295,000 maternal deaths occur during pregnancy, childbirth, and the immediate postpartum period each year, as well as 2 million stillbirths and 2.5 million neonatal deaths ^1– 3^. Ensuring universal access to good-quality care for all women during pregnancy, childbirth and the postpartum period would prevent the vast majority of these deaths ^4– 6^. The World Health Organization (WHO) produces evidence-based global guidelines to help health services, clinicians and communities ensure that the best care can be provided to pregnant women, regardless of where they give birth. Since 2017, the WHO Department of Sexual and Reproductive Health and Research has embarked on a “living guidelines” approach to update recommendations in maternal and perinatal health ^7^. Based on this approach, WHO’s portfolio of over 400 maternal and perinatal health recommendations is regularly assessed by an independent international panel of experts, to identify which recommendations are in most urgent need of updating, and if new recommendations are needed.

Developing and updating WHO recommendations for global use involves explicit consideration of available evidence for a given intervention across several criteria, including: the balance of benefits and harms, how stakeholders value different health outcomes, acceptability, feasibility, equity and cost-effectiveness of the intervention ^8^. Even when there is clear evidence that an intervention is beneficial, acceptable and feasible, policy makers must consider the resource implications of implementation at scale. Health budgets are finite and limited, meaning that adding (or expanding access to) an intervention has an opportunity cost that may result in detrimental reduction of another health intervention. In these instances, evidence on the affordability and cost-effectiveness of the intervention is critical to inform decision-making. The effectiveness evidence for a majority of WHO maternal and perinatal health recommendations are drawn from systematic reviews of randomised trials, however these reviews do not routinely evaluate outcomes related to resource needs or cost-effectiveness ^7^.

There have been previous efforts to map economic evaluations across different maternal health interventions, though these have been narrowly focused on selected interventions. For example, a 2013 scoping review identified 36 studies on economic benefits of reproductive, maternal, newborn and child health interventions, but it was limited to cost-benefit studies from low- and middle-income countries (LMICs) only, excluded studies published before 2000, and did not consider all maternal and perinatal interventions recommended by WHO ^9^. The 2016 Disease Control Priorities summarised cost-effectiveness evidence for selected, high-value maternal interventions, identifying 26 studies ^10^. More recently, systematic reviews of economic evaluations have been conducted for single interventions as part of WHO recommendation updates ^11, 12^. Other reviews have focused on economic evaluations of certain categories of interventions in LMICs, such as health systems strengthening strategies, or programs to increase utilisation and provision of care ^13, 14^.

A broad, contemporary synthesis of economic evaluations across a wide range of interventions would provide a critical resource for future updates of WHO maternal health recommendations. It could also provide a consolidated, navigable resource for policy makers, health managers, and clinicians to identify and consider evidence for decision-making in maternal health, including judgements around allocative efficiency and costing models for maternal health budgets ^15, 16^. Such a synthesis needs to be amenable to regular updating to reflect future changes in the underlying literature. Therefore, the aim of this project was to conduct a scoping review of primary economic evaluations of maternal health interventions to create such a database and to provide preliminary insights into the body of research conducted in this field.

## Methods

A systematic scoping review was undertaken in this study. A scoping review is a type of research synthesis that aims to map literature on a particular topic or research area, providing an opportunity to identify types and sources of evidence to inform practice, policymaking and research ^17^. This methodology was selected as we were seeking to examine the extent, range and nature of evidence on maternal health interventions and identify gaps in the literature, and not to formally summarise or pool data on cost-effectiveness of any single intervention ^18^. This review was conducted in line with the Levac *et al.* scoping review framework ^19^, which is an extended version of the Arksey and O’Malley framework ^20^, and the PRISMA Extension for Scoping Reviews (PRISMA-ScR) reporting checklist (extended data E5) ^18^. These frameworks help to ensure a consistent, thorough approach to the methodology of the review, and promote replicability. This protocol was registered and published on Open Science Framework (OSF) website ^21^.

### Eligibility criteria

For this review, we considered only full economic evaluations – including cost-benefit analyses, cost-effectiveness analyses, and cost-utility analyses – to be eligible ( Box 1). Studies with cost effectiveness data within, or alongside, randomised controlled trials of effectiveness were eligible. Systematic reviews of economic evaluations were not included. As this review focused on maternal health interventions, the population of interest was women who were pregnant or recently pregnant, in any stage of labour or childbirth, or in the postpartum period (up to 42 days). This review considered any intervention primarily aimed at improving maternal and perinatal health outcomes. This included any clinical, pharmacological, procedural, educational, or behavioural intervention implemented at any level (including individual, health care provider, community, facility, subnational or national levels). Pre-conception interventions, abortion-related interventions, interventions related to management of miscarriage or ectopic pregnancies, and interventions aimed only at newborns were not included.

**Box 1. Definitions of types of economic evaluations used for this review**

**Cost-benefit analysis (CBA)**

Economic evaluations in which the cost of the intervention is related to a value of benefits that uses a common or equal unit of measure, typically monetary.

**Cost-utility analysis (CUA)**

Economic evaluations in which the cost of the intervention is related to a multidimensional measure of effectiveness which considers not only the outcomes but the valuation of benefits, i.e. a measure of utility such as QALYs or DALYs.

**Cost-effectiveness analysis (CEA)**

Economic evaluations in which the cost of the intervention is related to a single clinical or natural measure of effectiveness, e.g. deaths, cases.

Adapted from: U.S. National Library of Medicine - Health Economics Information Resources: A Self-Study Course (Module 4)

Studies were eligible regardless of what comparator was used and considered any perspective (including societal or health system perspectives). They were eligible if they reported any quantifiable health outcome alongside costs, though the key outcomes of interest were cost-benefit outcomes (where health effects are valued in monetary terms), cost per quality-adjusted life year (QALY) or disability-adjusted life year (DALY), and cost per condition averted or life saved. Eligible studies were those published in peer-reviewed journals conducted in any country. We excluded records published as letters, editorials, or conference abstracts. No language restrictions were applied; for studies published in languages other than English an initial translation was carried out using open-source software (Google Translate) for assessing eligibility. If the study was potentially eligible and this translation was inadequate for data collection, we sought assistance from multilingual colleagues.

### Information sources and search strategy

We searched both specialist health economics databases (NHS Economic Evaluation Database and EconLit) and general medical and health databases (PubMed, Embase, CINAHL and PsycInfo) on 20 November 2020. For the period up to 2014, we limited searching to NHS EED, which provides access to over 17,000 economic evaluations of health and social care interventions. NHS EED collated results from weekly searches of MEDLINE, Embase, CINAHL, PsycInfo and PubMed until the end of December 2014. Economic evaluations added to NHS EED compare the costs and outcomes of two or more interventions using cost-benefit, cost-utility or cost-effectiveness analyses. NHS EED is available online but has not been updated since March 2015. Hence, for the period 2015 to 2020, we searched PubMed, EconLit, Embase, CINAHL and PsycInfo. The search strategies for these sources combine terms relevant to maternal health with terms related to economic evaluations (see extended data E1). Search terms for maternal health were derived from search strategies used by Cochrane Pregnancy and Childbirth to maintain and update their specialised register. Search terms for economic evaluations were derived from the search strategies used to populate NHS EED.

In consultation with an information specialist, we adopted a multi-phase approach to searching and screening records from PubMed. Phase 1 of the search was limited to records indexed with the most relevant MeSH term (Cost-Benefit Analysis). Phase 2 extended this to records indexed with other MeSH terms related to economics and costs. Phases 3a and 3b used free-text terms in the title/abstract limited to records not MeSH-indexed (i.e., the non-MEDLINE subset of PubMed). Phase 4 combined MeSH terms and free-text terms across all of PubMed. For pragmatic reasons, we adopted a sampling approach for the 16,135 unique records retrieved by phase 3b and phase 4 of the search, since we expected very few of these records to be relevant. We screened a 10% and 5% sample of phase 3b and phase 4, respectively. We similarly screened a 10% sample of 1025 NHS EED records obtained using non MeSH-indexed terms. Screening these sample records resulted in less than the pre-specified threshold of 3% being included in the review. Searches of Embase, CINAHL and PsycInfo were limited to records indexed with the appropriate subject indexing terms only. We also searched the WHO Global Health Library for any economic evaluations not identified from searches of the sources listed above. 

### Study selection, data extraction and analysis

Titles and abstracts of all identified citations were deduplicated in EndNote and imported into Covidence software for screening. Two review authors independently assessed unique citations against the eligibility criteria. Potentially relevant articles were included for full text review and assessed for eligibility by two independent authors. At both stages, disagreements were resolved through discussion or consulting a third author. Where more than one paper reported on the same study (i.e. using the same sample and methods), the papers were collated to ensure the primary study was the unit of interest ^22^.

Data extraction was conducted using a customised spreadsheet in Google Sheets. We extracted data on study characteristics, including: year, country, population of interest, period of intervention, context of care, intervention and comparator description, category of intervention, intervention mechanism, outcome measures, evaluation type and perspective, relation to WHO recommendation(s), cost year, currency, and data source. Country income levels were coded using World Bank data. We developed operational definitions for consistent coding of the extracted data (extended data E2). When coding the intervention mechanism of included studies, we used the Cochrane Effective Practice of Care (EPOC) classifications for health systems interventions. For each study, we searched the WHO website to identify whether the intervention or comparator considered by that study had a current WHO recommendation (for or against). If only part of the intervention was related to a recommendation (for example, when the study explored a package of interventions, of which one was a WHO-recommended intervention), that study was classified as partially linked to a WHO recommendation. All data were extracted by a single author, with a 15% sample independently reviewed by a second author. We conducted a series of consistency and validation checks for additional quality assurance. As this was a scoping review, no quality assessments of individual studies were performed. We reported findings on extracted variables using descriptive analysis with frequency tables and graphs on characteristics and coded categories of included studies as described above.

## Results

**Figure 1.**
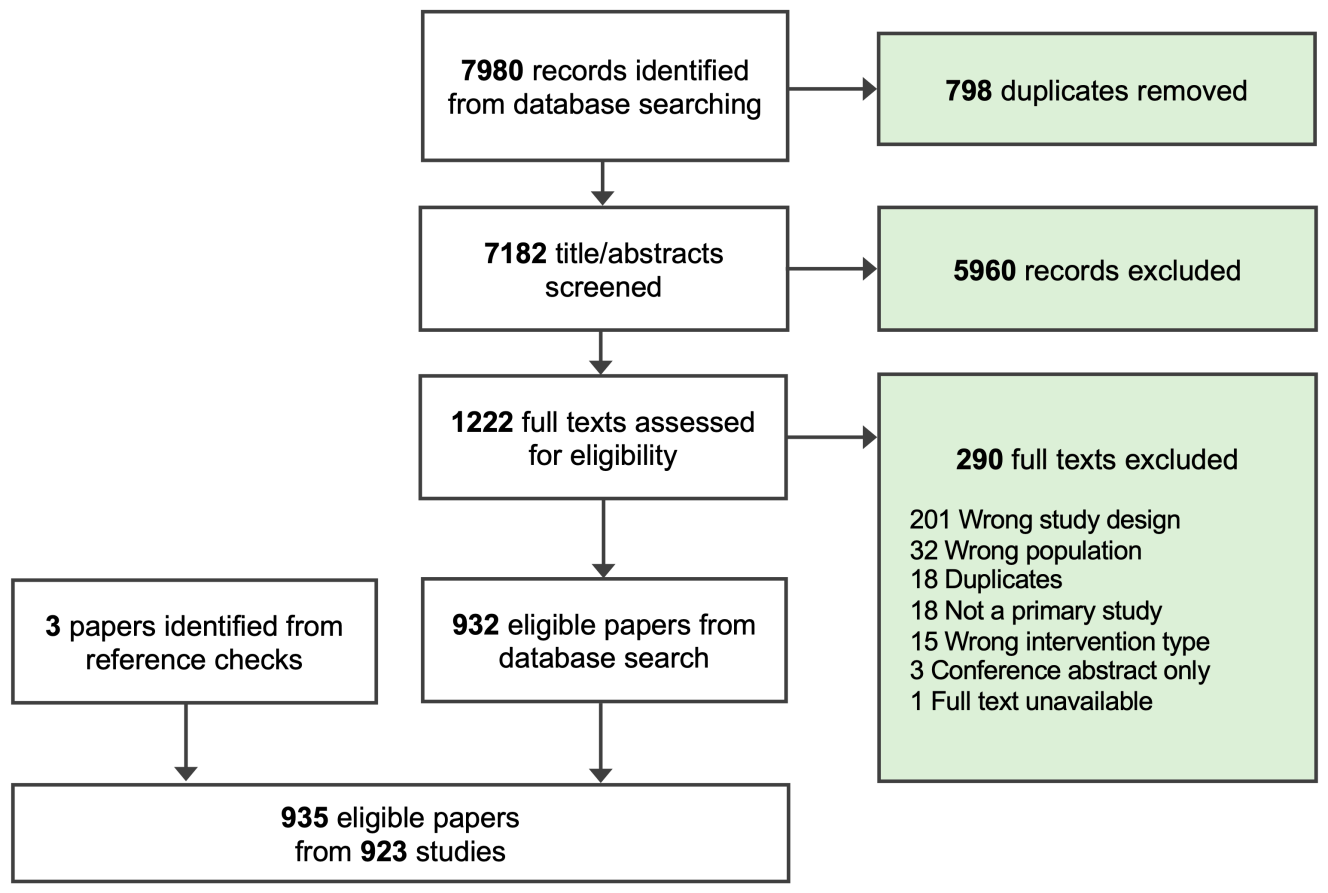
PRISMA flowchart of screening process.

**Figure 2.**
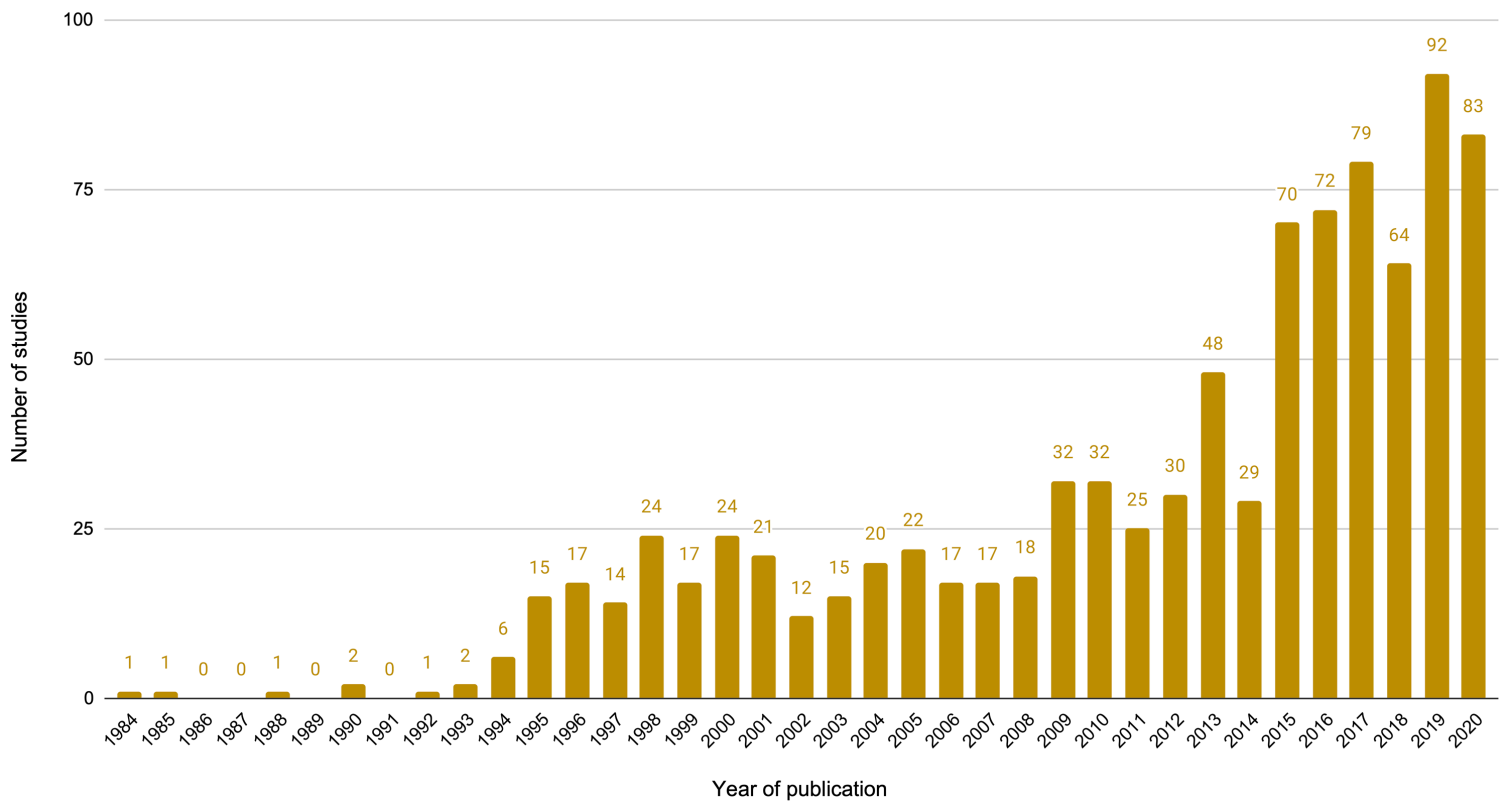
Number of studies by year of publication.

We identified 923 studies for inclusion in this review ( Figure 1). The number of economic evaluations of maternal health interventions has increased over time, with over half of all included studies (489 studies, 53.0%) published from 2014 onwards, compared to those from 1984 to 2013 (434 studies, 47.0%) and just over a quarter of studies (239 studies, 25.9%) in the last three years (2018-2020) ( Figure 2).

### Geography and income level

The economic evaluations were conducted in 72 countries (extended data E3: Table S1 ^23^). Ten countries (United States of America [USA], United Kingdom [UK], Canada, Australia, Netherlands, China, South Africa, India, France, and Spain) accounted for nearly 70% (642 studies) of all studies ( Table 1). The highest number of studies were from USA (313 studies), followed by the UK (119 studies), Canada (40 studies), and Australia (39 studies); 48 of the 72 countries had 5 or less studies. In total, 71.8% (663 studies) were conducted in high-income countries, with a further 21.3% (197 studies) in LMICs. The remaining 6.8% (63 studies) were conducted in multiple countries across different income levels ( Table 2). LMICs with the highest number of studies were China (24 studies), South Africa (23 studies), and India (17 studies).

**Table 1.** Top ten countries by number of included studies.

Rank	Country	World Bank income level	Number of studies	Percentage of total studies
1	United States of America	High income	313	33.9%
2	United Kingdom	High income	119	12.9%
3	Canada	High income	40	4.3%
4	Australia	High income	39	4.2%
5	Netherlands	High income	38	4.1%
6	China	Upper middle income	24	2.6%
7	South Africa	Upper middle income	23	2.5%
8	India	Lower middle income	17	1.8%
9	France	High income	16	1.7%
10	Spain	High income	13	1.4%
	*Total of all top 10 countries*		*642*	*69.6%*

**Table 2.** Number and proportion of included studies by country income level.

Country income level	Number of studies	Percentage of total studies
**High income**	**663**	**71.83%**
**Low and middle income**	**194**	**21.02%**
Upper middle income	89	9.64%
Lower middle income	63	6.83%
Low income	42	4.55%
**Multiple Total**	**66**	**7.15%**

### Population, intervention period, and setting

**Figure 3.**
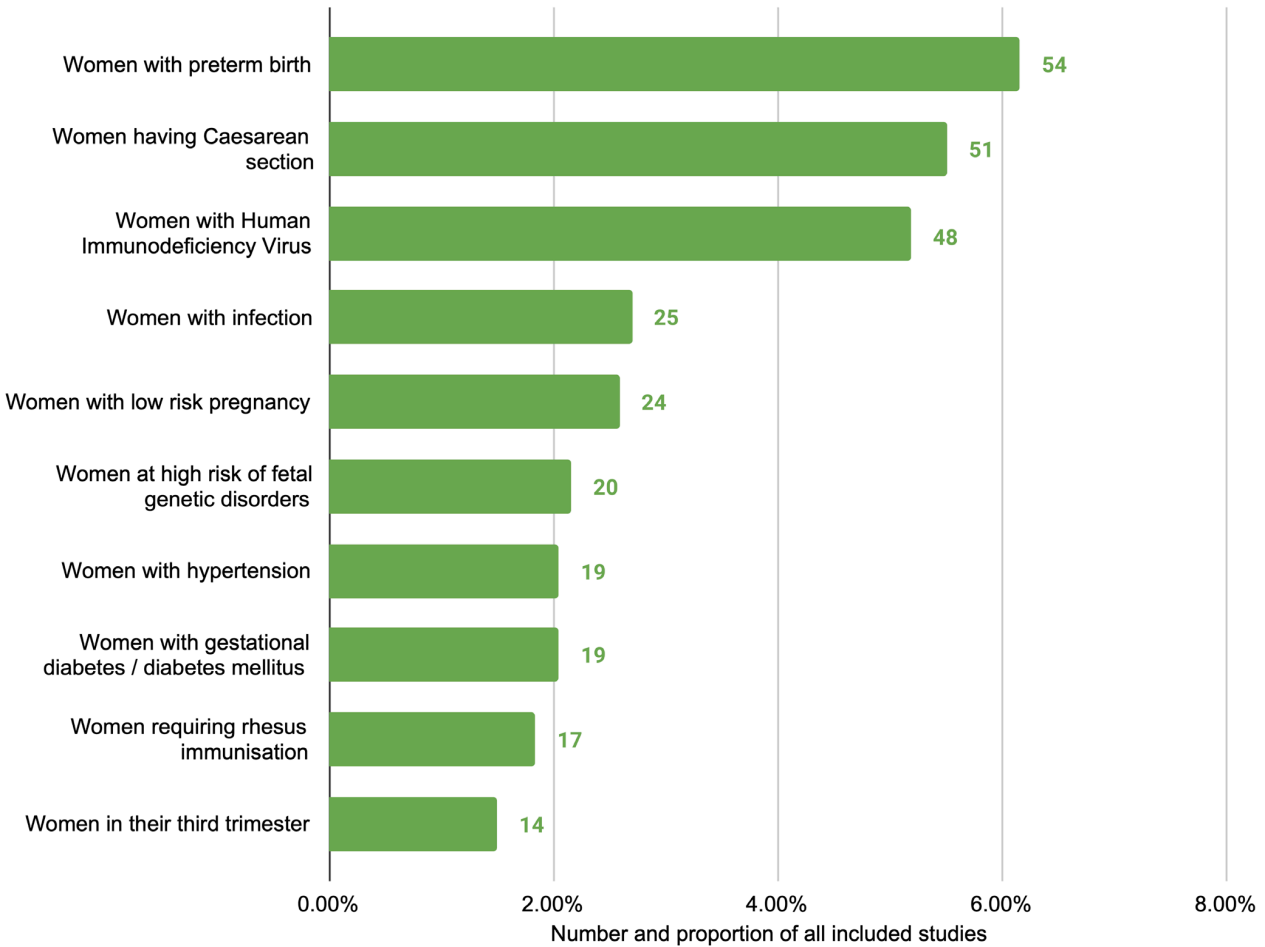
Top ten subpopulations of interest, excluding ‘all pregnant women and mothers’, by number of studies.

**Figure 4.**
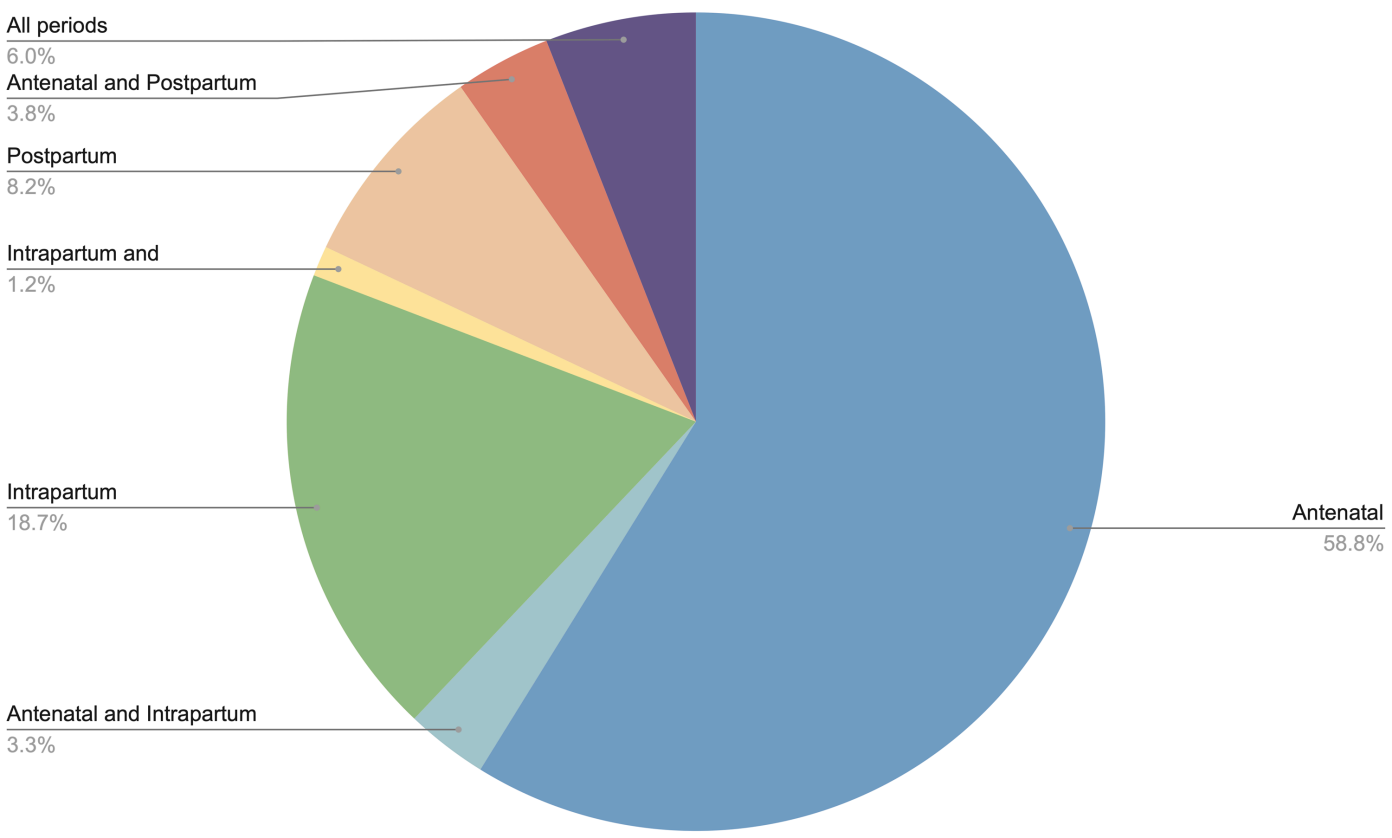
Number of studies by time period of intervention.

Studies varied in the population of interest they focused on. We categorised studies based on the subpopulation of interest and identified 53 subgroups (extended data E3: Table S2 ^23^). The most common were studies of women at risk of or experiencing preterm birth (57 studies), women undergoing caesarean section (51 studies) and women with HIV (48 studies) ( Figure 3). Approximately half (465 studies, 50.4%) broadly considered any or all pregnant women or mothers, without specific restrictions or focus. More than half of studies related to interventions only in the antenatal period (543 studies, 58.8%), followed by the intrapartum period only (173 studies, 18.7%), and the postpartum period only (76 studies, 8.2%); the remainder were a combination of two or more periods ( Figure 4). In terms of care setting, studies relating to outpatient services were most common (424 studies, 45.9%), followed by inpatient (224 studies, 24.3%), and a combination of both (147 studies, 15.9%) ( Table 3). Only 115 studies (12.5%) related to interventions outside of healthcare settings, including community, home-based, or telemedicine interventions.

**Table 3.** Number of studies by setting of care.

Setting of care	Number of studies	Percentage of total studies
Outpatient facility	424	45.9%
Inpatient facility	224	24.3%
Both inpatient and outpatient facility	147	15.9%
Community	42	4.6%
Home-based	32	3.5%
Population-based	20	2.2%
Telemedicine	18	2.0%
Multiple	13	1.4%
Unspecified	3	0.3%
*Total*	*923*	*100.0%*

### Intervention categories and mechanisms

We identified 61 distinct categories of interventions, which we mapped to 10 broad topic areas (extended data E3: Table S3 ^23^). The most common studies were those addressing prevention, recognition, and management of infection not specific or exclusive to pregnancy, such as HIV, Group B Streptococcus (GBS), and Hepatitis B (221 studies, 23.9%); labour and childbirth care (e.g. caesarean section) (157 studies, 17.0%); prevention, diagnosis, and management of obstetric complications (145 studies, 15.7%); screening and diagnosis of genetic disorders (109 studies, 11.8%); models of care (e.g. midwifery-led care) (103 studies, 11.2%), and routine antenatal and postpartum care (77 studies, 8.3%) ( Table 4). In assessing interventions, we also identified 52 intervention mechanisms mapped to seven broad types (extended data E3: Table S4 ^23^). The three most common were clinical interventions (379 studies, 41.1%), diagnostic tests (338 studies, 36.6%), and health system delivery arrangements (97 studies, 10.5%).

**Table 4.** Number and proportion of studies for broad categories of maternal health interventions.

Broad category of maternal health intervention	Number of studies	Percentage of total studies
**Non-obstetric infection - prevention, recognition and management** (e.g. Human Immunodeficiency Virus, Group B Streptococcal infection, Hepatitis B)	221	23.9%
**Labour and childbirth care** (e.g. premature labour, induction of labour, caesarean section)	157	17.0%
**Obstetric complications - prevention, recognition and management** (e.g. obstetric haemorrhage, diabetes, hypertensive disorders)	145	15.7%
**Detection of genetic disorders** (e.g. Trisomy 13/18/21 screening)	109	11.8%
**Models of care** (e.g. midwifery-led care)	103	11.2%
**Routine antenatal and postpartum care** (e.g. vaccinations, nutrition, breastfeeding promotion)	77	8.3%
**Medical complications of pregnancy and postpartum - prevention,** ** recognition and management** (e.g. mental health, anaemia, embolism)	41	4.4%
**Foetal and neonatal health** (e.g. congenital anomalies)	38	4.1%
**Lifestyle and behavioural** (e.g. smoking cessation, promotion of physical activity during pregnancy)	26	2.8%
**Prevention of pregnancy loss**	3	0.3%
**Multiple**	3	0.3%
*Total*	*923*	*100.0%*

### Relation to WHO recommendations

**Figure 5.**
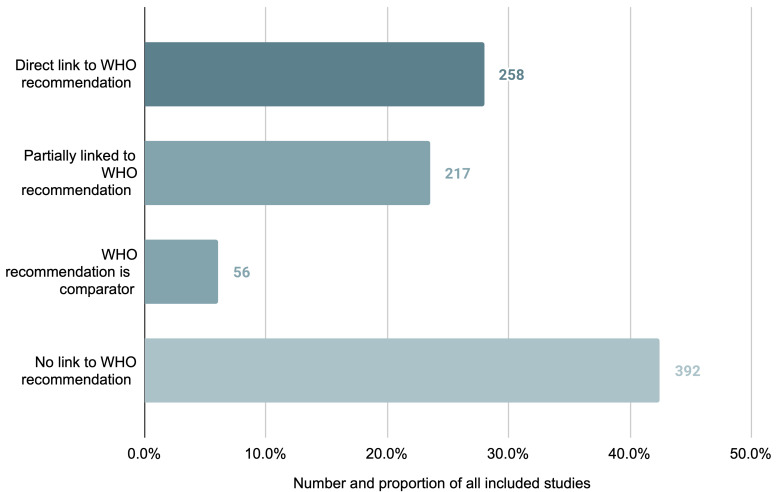
Number and proportion of studies per identified relation to corresponding WHO recommendation for the studied intervention.

Of the 923 studies in the review, 531 (57.5%) studies assessed an intervention or comparator related to a published WHO recommendation. For 258 studies (27.9%) the intervention was directly linked; for 217 studies (23.6%) the intervention was only partially linked; and for 56 studies (6.1%) the comparator was linked. A total of 392 studies (42.5%) assessed interventions and comparators for which there is no current WHO recommendation ( Figure 5). Within the 258 studies where the intervention was directly linked to a current WHO recommendation, the most frequent interventions related to HIV management in pregnancy (54 studies); obstetric haemorrhage (23 studies); midwifery-led care (14 studies); syphilis in pregnancy (14 studies) and induction of labour (11 studies). Of those studies exploring interventions which were not the subject of a current WHO recommendation, categories including genetic screening (58 studies); premature labour/preterm birth (48 studies); vaccination in pregnancy (26 studies); caesarean section (23 studies); and Group B streptococcal disease (17 studies) were most common (extended data E3: Table S5 ^23^).

### Type of economic analysis

Cost effectiveness analyses (CEA) using condition or intervention-specific measures of health effects accounted for more than half of all included studies (573 studies, 62.1%). 13 studies (1.4%) conducted a cost-benefit analysis, valuing health effects in monetary terms, and 337 studies (36.5%) conducted cost-utility analysis (CUA) valuing health effects using quality-of-life measures. Within the CUA studies, studies conducted in high-income countries primarily assessed quality-adjusted life years (QALYs) (206/209 studies) while those in LMICs primarily assessed disability-adjusted life years (DALYs) (61/90 studies), and the remaining CUA studies were conducted across multiple income levels. Included studies considered seven different self-reported cost-effectiveness perspectives, with some studies reporting more than one perspective ( Table 5). Of the seven perspectives, health sector was the most reported (205 studies, 22.2%), followed by societal (176 studies, 19.1%), provider (98 studies, 10.6%), government (96 studies, 10.4%), third party funder (41 studies, 4.4%), payer unspecified (30 studies, 3.3%), and finally the patient (20 studies, 2.2%). Nearly one-third of studies did not specify the perspective used (302 studies, 32.7%).

**Table 5.** Number of studies within the dataset that self-report one of the seven identified cost-effectiveness perspectives.

Cost-effectiveness perspective	Number of studies	Percentage of total studies *
Health sector	205	22.2%
Societal	176	19.1%
Provider	98	10.6%
Government	96	10.4%
Third party funder	41	4.4%
Payer (unspecified)	30	3.3%
Patients	20	2.2%
No perspective reported	302	32.7%

*Studies reporting more than one perspective are listed against each applicable perspective; as such, the percentages are not cumulative.

## Discussion

### Key findings and interpretation

This review identified and categorised 923 economic evaluations of maternal health interventions published over a 37-year period (the earliest study identified was from 1984). To our knowledge, this is the first such broad mapping of economic evaluations of interventions used during pregnancy, childbirth, and the postpartum period. The number of maternal economic evaluations have increased markedly in the last decade, with over half of included studies published since 2014. Included studies used diverse methods to explore a wide range of interventions, and the majority of studies presented evidence from high-income countries.

Comparison with other reviews of economic evaluations in maternal health similarly found that research in this area has increased. Previous reviews typically had a narrower focus, including studies focused on a single or specific set of interventions or evidence from specific settings ^9– 14^, and consequently identified a smaller number of eligible studies (typically less than 30). For example, a 2018 systematic review of health systems strengthening economic evaluations in maternal and perinatal health identified 24 eligible studies, 23 of which were published since 2000 ^13^. A 2014 review identified 48 economic evaluation studies on utilisation and provision of maternal and newborn care in LMICs, of which 36 were published since 2000 ^14^. These reviews, along with the upward trend of publications identified in our review, suggest increasing demand for economic evaluations in this topic area.

Evidence from economic evaluations can be difficult to generalise across different settings, given differences in health system arrangements, payment models, and labour, equipment and medicine costs between jurisdictions ^24^. Global estimates of maternal and neonatal mortality rates show that the vast majority of these deaths occur in LMICs ^1– 3^. In these contexts, health budgets are likely to be more limited, with difficult decisions to be made about which interventions to prioritise when resources are scarce. Affordability is also likely to be an issue for those countries where individuals and families are often required to cover the cost of healthcare (i.e. out-of-pocket costs). Despite these public health realities, this review found most economic evaluations were conducted in high-income settings; only 21% of included studies were set in LMICs, and seven high-income countries accounted for nearly two-thirds of available economic evidence. This is consistent with a 2013 scoping review of cost-benefit analysis studies pertaining to reproductive, maternal, newborn and child health in LMICs, which identified only 36 eligible studies ^9^. Larger health budgets in high-income countries may be a driver for this, creating a stronger incentive to ensure value for money across higher overall health expenditure. The breadth of healthcare interventions available in high-income settings may also incentivise health economic research since there are more options to be considered by policymakers and insurers when allocating budgets. Nevertheless, this inequity in health economic research suggests efforts need to be better targeted to settings and health systems where the mortality and morbidity burden is greatest. Barriers to implementation of effective interventions in these settings are complex and diverse, but often include economic factors ^25^. Greater investment in health economic evaluations for LMIC contexts – tailored specifically to the interventions used in these settings – would probably improve policy decision-making in these settings, yielding additional public health benefits.

Studies considered a diverse range of interventions and patient sub-populations, such as women experiencing preterm birth, caesarean section, or HIV. However, a relatively small proportion of included studies related to the leading causes of maternal deaths globally ^26^. Specifically, only 37 studies (4.0% of all studies) focused on obstetric haemorrhage, 33 studies (3.6%) on hypertensive disorders, 26 studies (2.8%) on infections that could lead to sepsis, and 7 studies (0.8%) on embolism – these four conditions comprise the leading direct causes of global maternal deaths. In this review, the most frequently studied interventions related to genetic screening and diagnostic tests (including for cystic fibrosis, trisomy disorders, and thalassaemia traits) (109 studies, 11.8%), HIV in pregnancy (including prevention of maternal-to-child transmission) (82 studies, 8.9%), preterm labour and birth (64 studies, 6.9%), and diabetes in pregnancy (38 studies, 4.1%). This may be related to the large proportion of studies conducted in high-resource settings, where maternal deaths are comparatively rare and economic research priorities may lie elsewhere ^27^.

When developing their recommendations, WHO prioritises interventions that are likely to have the greatest impact on reducing global maternal mortality and morbidity – as well as increasing the experience and wellbeing of women – and cost-effectiveness is a key consideration in developing these recommendations ^8^. This review identified 258 studies that provide cost-effectiveness evidence on interventions directly linked to current WHO recommendations (extended data E3: Table S9 ^23^). However, the majority of studies identified either did not relate (or relate only partially) to a WHO recommendation. This similarly suggests a dearth of economic evaluation research on those maternal health interventions of highest global priority.

### Strengths and limitations

This scoping review used a robust search in multiple databases, allowing us to identify a large number of studies across a broad range of interventions, settings and analytical designs. Adherence to the Levac *et al.* scoping review methodological framework ^19^ and PRISMA-ScR checklist ^18^ maintained consistency in our approach, while quality assurance and validation checks ensured data accuracy. Despite our best efforts, it is possible that some eligible studies were not captured. For example, while effectiveness trials may report on cost outcomes, this may not be clearly documented in the study abstract or main findings, making it difficult to detect. We also relied upon the NHS EED database to identify studies published before 2015. While our search from 2015 onwards focused on the same databases indexed by NHS EED, we are unable to fully assess the veracity of their eligibility assessment process and whether the two approaches meaningfully differed. An additional challenge in this review was in systematically classifying the population, intervention, comparators, and outcomes used across studies. For example, economic evaluations may involve the same target population, but report on different outcomes of interest, or consider different cost perspectives. With the data extracted in this review, we were not able to explore some economic analytical questions of public health importance (e.g. any differences in study findings across private vs public contexts), however, future expansion of this scoping review may allow us to do so. 

### Future research and implications for practice

This review was conducted to support WHO activities on living guidelines in maternal health ^7^. In light of future updates to those guidelines, we intend to regularly update this review. In future updates, we anticipate incorporating quality assessments for individual studies that are generated from evidence syntheses of specific interventions, though there are acknowledged limitations in available tools for assessing quality of health economic literature ^28^. The identification of studies in this review can be useful to maternal health guideline development or policy decision-making processes by providing a searchable, contemporary database of health economic evidence. This can be used to identify all available studies for specific interventions, subpopulations, and contexts. Further to this, the gaps in maternal economic evaluations that have been identified in this review can provide insights into where future research needs to be targeted.

## Conclusion

We identified 923 economic evaluations of maternal health interventions, covering a wide range of subpopulations of women and health conditions. While the volume of economic evaluations has increased over time, there are significant disparities between available economic literature, and the causes and settings of maternal and newborn deaths. Future health economic research needs to focus on interventions to address the major drivers of maternal morbidity and mortality, and their implementation in limited-resource contexts. The review findings provide a comprehensive, and navigable resource for economic evidence to support maternal health guideline and policy development.

## Data availability

### Underlying data

All data underlying the results are available as part of the article and no additional source data are required.

### Extended data

Zenodo: Economic evaluations of maternal health interventions: a scoping review (extended data repository). https://doi.org/10.5281/zenodo.6030745 ^23^

This project contains the following underlying data:

- Economic evidence in MH - Extended data E1 Search strategies.docx
- Economic evidence in MH - Extended data E2 Operational definitions.docx
- Economic evidence in MH - Extended data E3 Additional results tables.docx
- Economic evidence in MH - Extended data E4 Extracted data for included studies.docx
- Economic evidence in MH - Extended data E5 PRISMA-ScR checklist.docx
- LICENSE.txt

Data are available under the terms of the Creative Commons “Attribution 3.0 IGO” data license (CC BY 3.0 IGO).
