# Supplementary material for: Economic evaluations of maternal health interventions: a scoping review
Source: F1000Res. 2023 May 16;11:225. Originally published 2022 Feb 24. [Version 2] doi: 10.12688/f1000research.76833.2 (PMC11420617; doi:10.12688/f1000research.76833.2)
Supplement: Peer review history

# Reviewer report: Reviewer response for version 2

**DOI:** 10.5256/f1000research.148031.r320174

**Published:** 2024-09-23

**Authors:** Sameera Senanayake

**Affiliations:** 1 Queensland University of Technology (QUT), Musk Ave, Kelvin Grove, QLD, Australia

The manuscript titled *"Economic evaluations of maternal health interventions: a scoping review"* provides an extensive review of economic evaluations focused on maternal health interventions. The review aims to support global maternal health guideline development, particularly by the World Health Organization (WHO), through the synthesis of 923 economic evaluations conducted across 72 countries.

 The manuscript is well-written and clearly structured, making it easy for readers to follow the objectives and findings. The authors have effectively articulated their methodology and results, providing valuable insights into the state of economic evaluations for maternal health interventions.

 However, there are a few areas for improvement. The search was conducted in November 2020, meaning that more recent studies may not have been included. Given ongoing advancements in maternal health interventions, it would be important to update the search to include the latest evidence, if possible, to strengthen the paper's relevance.

 Additionally, while the study justifies not applying a CHEERS quality assessment due to its scoping nature, including a quality assessment would have enhanced the robustness of the findings. I understand that reassessing more than 900 articles for quality may not be practically feasible at this stage, but it would have been valuable if this had been included from the outset.

Are the rationale for, and objectives of, the Systematic Review clearly stated?

Yes

Is the statistical analysis and its interpretation appropriate?

Yes

If this is a Living Systematic Review, is the ‘living’ method appropriate and is the search schedule clearly defined and justified? (‘Living Systematic Review’ or a variation of this term should be included in the title.)

Not applicable

Are sufficient details of the methods and analysis provided to allow replication by others?

Yes

Are the conclusions drawn adequately supported by the results presented in the review?

Yes

Reviewer Expertise:

Economic evaluations, Choice experiments (discrete choice experiments)

I confirm that I have read this submission and believe that I have an appropriate level of expertise to confirm that it is of an acceptable scientific standard.

# Reviewer report: Reviewer response for version 2

**DOI:** 10.5256/f1000research.148031.r173802

**Published:** 2023-07-14

**Authors:** Malte Sandner

**Affiliations:** 1 Department of Economics, Nueremberg Institute for Technology, Nürnberg, Germany

I value the effort of the autors to improve the article.

Are the rationale for, and objectives of, the Systematic Review clearly stated?

Yes

Is the statistical analysis and its interpretation appropriate?

Not applicable

Are sufficient details of the methods and analysis provided to allow replication by others?

Yes

Are the conclusions drawn adequately supported by the results presented in the review?

Partly

Reviewer Expertise:

Labour, Education, Family, Health Economics

I confirm that I have read this submission and believe that I have an appropriate level of expertise to confirm that it is of an acceptable scientific standard.

# Reviewer report: Reviewer response for version 1

**DOI:** 10.5256/f1000research.80813.r163254

**Published:** 2023-03-24

**Authors:** Malte Sandner

**Affiliations:** 1 Department of Economics, Nueremberg Institute for Technology, Nürnberg, Germany

This article gives an overview about economic evaluations of maternal health interventions. The article includes 923 studies which meet the criteria applied by the researchers. These criteria are overall comprehensible and I guess the most relevant studies are included in the review. 

 I think the authors should make clearer why the review is relevant. I think one reason for the relevance is to identify areas in which little research is conducted and where more research is necessary. I think the article is so far neglecting this aspect. 

 Furthermore, the article should formulate certain areas where more research is needed. The authors go into this direction, but I think they can identify more particular gaps by interacting setting of care, subpopulations, country, country income level, and broad categories of maternal health interventions.

 Finally, I think the broad categories of maternal health interventions can be explained better. On which base are these broad categories chosen? How is the categorization conducted? I can imagine many interventions can also put in more than one broad categories of maternal health interventions.

Are the rationale for, and objectives of, the Systematic Review clearly stated?

Yes

Is the statistical analysis and its interpretation appropriate?

Not applicable

Are sufficient details of the methods and analysis provided to allow replication by others?

Yes

Are the conclusions drawn adequately supported by the results presented in the review?

Partly

Reviewer Expertise:

Labour, Education, Family, Health Economics

I confirm that I have read this submission and believe that I have an appropriate level of expertise to confirm that it is of an acceptable scientific standard, however I have significant reservations, as outlined above.

# Response

**Published:** 2023-05-10

**Authors:** Katherine Eddy

**Affiliations:** Macfarlane Burnet Institute for Medical Research and Public Health, Australia

***Reviewer comment 1: **"This article gives an overview about economic evaluations of maternal health interventions. The article includes 923 studies which meet the criteria applied by the researchers. These criteria are overall comprehensible and I guess the most relevant studies are included in the review."*

**Response:** Thank you for your comment.

***Reviewer comment 2: **"I think the authors should make clearer why the review is relevant. I think one reason for the relevance is to identify areas in which little research is conducted and where more research is necessary. I think the article is so far neglecting this aspect. Furthermore, the article should formulate certain areas where more research is needed. The authors go into this direction, but I think they can identify more particular gaps by interacting setting of care, subpopulations, country, country income level, and broad categories of maternal health interventions."*

**Response: **Thank you for your feedback, we agree that evidence mapping is a key reason for the review’s relevance and have included an amendment to the Introduction section highlighting this. We likewise agree that an important finding of the review is that there are gaps in the existing evidence base for economic evaluations of maternal health interventions. The discussion highlights the need for further research in low and middle-income countries as a matter of priority, and also suggests that there should be greater focus on interventions that address the key causes of maternal mortality and interventions that are the subject of a WHO recommendation. Further to this, we have added discussion on which geographic regions and population subgroups would benefit from additional focus.

***Reviewer comment 3: **"Finally, I think the broad categories of maternal health interventions can be explained better. On which base are these broad categories chosen? How is the categorization conducted? I can imagine many interventions can also put in more than one broad categories of maternal health interventions."*

**Response: **The intervention categories and broad topic areas for maternal health interventions in this paper were developed inductively from the included studies through discussion with study authors. This was an iterative process, during which operational definitions were developed to ensure consistent coding of the extracted data. Following coding, categories were reviewed against the operational definitions to ensure consistency. The methods section has been amended to include further detail regarding this process. The operational definitions of each intervention category are set out in Extended Data E2: Table S3.

 Categorisation for each study was determined during the data extraction process, as described in ‘Study selection, data extraction and analysis’. The first reviewer categorised each study, on the basis of the described intervention within the study. A 15% sample of data extraction (including this categorisation) was independently reviewed by a second author. A series of consistency and validation checks were conducted, which further confirmed that studies of the same intervention appeared in the some broad category. 

 We acknowledge that some studies were relevant to more than one category of intervention, but that this would have resulted in double-counting in our analysis. Accordingly, we prioritised the most relevant category for each study for the purpose of analysis. An amendment to reflect this has been added to the limitations section.
